# Molecular Features and Methylation Status in Early Onset (≤40 Years) Colorectal Cancer: A Population Based, Case-Control Study

**DOI:** 10.1155/2015/132190

**Published:** 2015-10-19

**Authors:** Giulia Magnani, Daniela Furlan, Nora Sahnane, Luca Reggiani Bonetti, Federica Domati, Monica Pedroni

**Affiliations:** ^1^Dipartimento di Medicina Diagnostica, Clinica e Sanità Pubblica, Università di Modena e Reggio Emilia, 41124 Modena, Italy; ^2^Dipartimento di Chirurgia e Scienze Morfologiche, Università dell'Insubria, 21100 Varese, Italy; ^3^Divisione di Anatomia Patologica, Policlinico di Modena, 41124 Modena, Italy

## Abstract

Colorectal cancer is usually considered a disease of the elderly. However, a small fraction of patients develops colorectal cancer earlier. The aim of our study was to define the frequency of known hereditary colorectal syndromes and to characterise genetic and epigenetic features of early nonhereditary tumors. Thirty-three patients ≤40 years with diagnosis of colorectal cancer and 41 patients with disease at >60 years of age were investigated for MSI, Mismatch Repair proteins expression, *KRAS* and *BRAF* mutations, hypermethylation, and LINE-1 hypomethylation. Detection of germline mutations was performed in Mismatch Repair, *APC* and *MUTYH* genes. Early onset colorectal cancer showed a high incidence of hereditary forms (18%). *KRAS* mutations were detected in 36% of early nonhereditary tumors. Early onset colorectal cancer disclosed an average number of methylated genes significantly lower when compared to the controls (*p* = 0.02). Finally both of the two groups were highly methylated in *ESR1*, *GATA5*, and *WT1* genes and were similar for LINE-1 hypomethylation. The genetic make-up of carcinomas differs from young to elderly patients. Early onset tumors showed more frequently a constitutional defective of Mismatch Repair System and a minor number of methylated genes. Hypermethylation of *ESR1*, *GATA5*, and *WT1* genes suggests possible markers in the earlier diagnosis of colorectal tumorigenesis.

## 1. Introduction

Colorectal cancer (CRC) is the second most common cancer in both men and women in Western Europe, North America, Australia/New Zealand, and Japan, whereas it remains less frequent in Africa and Asia [1]. Colorectal cancer is usually considered a disease of the elderly in both sexes; data from Cancer Registries indicate that age-specific cancer incidence rises sharply after the age of 50–55 years and that mean age of affected individuals is around 70 years [2]. However, in a small fraction of patients (2-3% of all affected individuals), colorectal malignancies may develop earlier [3].

A review of the Surveillance Epidemiology and End Results (SEER) Program data from 2005 to 2009 provides more detailed information regarding colorectal cancer in younger patients suggesting that the incidence in younger patients is increasing along time (ages <20 to 54 years) [4], whereas other data would show a relative stability of the rates [5]. Part of these discrepancies can be attributed to the different age-limits proposed for the definition of early onset (or “juvenile”) tumors, which have been set at age 40, 45, or 50 depending on the authors and purposes of the studies [6, 7]. Although there is no clear age cut-off defined, the majority of studies classify patients diagnosed with cancer at age <40 years as “early onset” (or “young”) [8]. The reasons whereby some individuals develop colorectal cancer at an unusual age are poorly understood. Therefore, the etiology and biological characterisation of the majority of early onset colorectal neoplasms remain poorly defined.

From a molecular point of view, early onset tumors represent a heterogeneous group of diseases, including known hereditary syndromes, familial cases, and apparently sporadic colorectal cancer. Hereditary diseases explain only part of early onset colorectal cancer [9] and mainly include Lynch syndrome [7, 10–12], Familial Adenomatous Polyposis (FAP) [13], Peutz-Jeghers syndrome [14], Cowden syndrome/Bannayan [15], and* MUTYH*-associated polyposis (MAP) [13, 16].

Recent studies have suggested that the clinic-pathologic features related to early onset colorectal cancer [7] are more likely to be present at advanced stages, to be poorly differentiated, and to be located in the distal colon and in the rectum, although marked differences have never been reported. Morphologically, early onset CRCs more frequently display adverse histological features, such as signet ring cell differentiation and perineural and venous invasion [9]. Investigation of somatic profiles showed an increasing number of tumors with* KRAS*,* BRAF*, and* PIK3CA* oncogenes mutations related to advanced age. Mutations in the tumor suppressor genes* TP53* and* PTEN* were more frequent in the early onset group [17]. Other findings showed that young patients had significantly more chromosomal aberrations in their tumors than patients aged >70 years and microsatellite stable (MSS) colorectal cancer [7, 17, 18]. Recently, LINE-1 hypomethylation has been reported to be a distinct feature of MSS young age colorectal cancers (≤45 years), suggesting that the genomic hypomethylation may represent a possible pathway of early onset colorectal carcinogenesis [19]. By contrast, very few data are available about the frequency of CpG island methylator phenotype (CIMP) in early onset CRCs [19].

The knowledge about this clinically and genetically heterogeneous group of colorectal cancers remains limited and further studies are essential. To address this issue, we conducted a population based case-control study on CRCs diagnosed before the age of 40 years in order to define the frequency of known hereditary CRC syndromes in this subset of patients and to better characterize genetic and epigenetic features of early nonhereditary tumors compared with sporadic late onset CRCs.

## 2. Materials and Methods

### 2.1. Patients

The patients described in this study were recruited through the specialized Colorectal Cancer Registry of Modena in the period 1984–2008. In the 25 years of registration, we recruited 38 patients affected by adenocarcinoma in the colon-rectum diagnosed ≤40 years, which represented about 1% of the overall adenocarcinomas diagnosed (4,692 cases). We collected the biological material (blood sample, normal colorectal mucosa, and tumoral tissue) of 33 cases out of 38 and investigated the family history relative to the cases, including at least first-degree or second-degree relatives.

In order to compare some molecular features—including methylation of DNA—of early onset colorectal cancer with maturity onset disease, we selected a specific control group composed of 41 patients with late onset colorectal cancer (age-range: 61–85 years) that were matched to the study group for the following characteristics: sex, tumor location, and stage. Family cancer history was not present in late onset group. Tumors were classified as low-grade and high-grade adenocarcinoma [20] and staged with the Dukes' classification.

The study was approved by the local ethics committee. All individuals, or first-degree relative in case of death of the index case, gave their written consent for blood samples and tissue specimen analysis.

### 2.2. DNA Extraction

Genomic DNA from each patient was extracted by formalin-fixed, paraffin-embedded normal and tumor tissues. Serial sections from paraffin-embedded matched normal and neoplastic primary tissue were stained with Hematoxylin-eosin; representative normal and tumor regions were identified by microscopic examination. Areas of tumor tissue with more than 80% of malignant cells were selected in all cases, as previously described in detail [21]. Constitutional DNA from peripheral blood was obtained using QIAamp DNA Mini kit (Qiagen), according to the manufacturer's instructions.

### 2.3. MSI Analysis

MSI status of all tumors was evaluated using four fluorescent-labeled mononucleotide markers: BAT25, BAT26, NR24, and CAT25. These quasimonomorphic markers were selected after in-depth review of the literature for their very high sensitivity and specificity in identifying Mismatch Repair-deficient tumors [22–24]. The resulting panel composed of four markers reduced the time and cost involved in MSI testing. Other authors as Xicola and Deschoolmeester suggested a MSI analysis shortcut [23, 24]. Using this mononucleotide markers panel, a tumor was defined as MSI(+) when showing instability with at least three markers. MSI analysis was performed as described previously [21].

### 2.4. Immunohistochemical Analysis of MMR Proteins

Immunohistochemical (IHC) evaluation of MMR proteins expression was carried out on paraffin-embedded tissue sections of all tumors. The following mouse monoclonal antibodies were used: anti-MLH1, anti-MSH2 (PharMingen, San Diego, CA), and anti-MSH6 Transduction Laboratories, BD Biosciences, Brazil. For PMS2 protein, a rabbit monoclonal antibody was used (Ventana Roche Diagnostic, Italy). Immunostaining was executed by the avidin-biotin peroxidase technique; diaminobenzidine was used as a chromogen. Staining was carried out in NEX-ES Automatic Staining System, after counterstaining with Hematoxylin. Normal tissue and stromal cells or lymphocytes adjacent to the respective tumor were used as internal positive controls. Loss of MMR proteins expression was defined as complete absence of nuclear staining in tumor cells (but maintained in normal epithelial and stromal cells).

### 2.5. Analysis of Germline Mutations in* MMR*,* APC*, and* MUTYH* Genes

Cases showing MSI and lack of expression of one of MMR proteins in tumors were investigated to detect germline mutations in* MMR* genes. Patients with clinical features of Familial Adenomatous Polyposis were assessed for* APC *gene and all young patients were screened for the presence of* MUTYH* gene mutations. Analysis of germline mutations was performed by direct sequencing of the PCR products obtained using the Dye Terminator Cycle Sequencing Kit (CEQ DTCS Kit, Beckman Coulter) and reactions were run on a CEQ 8000 capillary sequencer (Beckman Coulter). To exclude the possibility of large genomic rearrangements in* MMR* genes, we used Multiplex Ligation-Dependent Probe Amplification (MLPA) procedure by SALSA P003-B2 kit (MRC-Holland, Amsterdam, Netherlands) and to confirm results SALSA P248-A2 kit (MRC-Holland). The entire open reading frame of* APC* gene was also analysed for the presence of deletions or rearrangements by using the SALSA P043 kit (MRC-Holland). Pathogenic mutations were detected twice and confirmed in a second blood sample of the patient.

### 2.6. Somatic* BRAF* and* KRAS* Mutations Analysis

All 74 tumors were analysed for* KRAS* and* BRAF *activating mutations. In* KRAS*, the more frequently mutated codons 12, 13, and 61 were analysed [25]. In* BRAF* gene, we amplified exon 15 which includes the hot spot for mutation codon 600 (V600E). Mutation analysis was performed by direct sequencing with the use of the standard protocol and running on Beckman Coulter CEQ 8000 instrument.

### 2.7. Methylation-Specific Multiplex Ligation-Dependent Probe Amplification (MS-MLPA) Analysis

MS-MLPA analysis was performed on all the 74 tumors (33 early onset CRC and 41 control cases) using the ME001 MS-MLPA Tumor Suppressor-1 Kit, the ME002 MS-MLPA Tumor Suppressor-2 Kit, and the ME011 MS-MLPA Mismatch Repair Genes Probemix Kit (MRC-Holland, Amsterdam, Netherlands). Using these three kits, a total of 38 tumor suppressor genes were analysed for aberrant promoter methylation. All these genes are frequently silenced by methylation in tumors of different sites, and they frequently harbour genetic alterations during tumorigenesis. Methylation-specific MLPA (MS-MLPA) is a semiquantitative method for methylation profiling. MS-MLPA is a variant of the MLPA technique in which copy number detection is combined with the use of a methylation-sensitive restriction enzyme [26]. Probe sequences, gene loci, and chromosome locations can be found at http://www.mlpa.com/. The experimental procedure was carried out according to the manufacturer's instructions. Reaction products were separated on an automated sequencer (ABI 310 capillary) and visualised with Genemapper analysis v.4.0 (Applied Biosystems). Values corresponding to peak size (base pairs) and peak height were used for further data processing by Coffalyser V7 software (MRC-Holland). All MS-MLPA reactions were performed at least two times. The methylation profile of each sample was assessed according to MRC-Holland instructions.

Aberrant methylation was scored as a categorical variable using a specific Methylation Ratio (MR) for each gene corresponding to the highest level of accuracy of the test, according to previously reported [27].

### 2.8. LINE-1 PCR and Pyrosequencing

The methylation status of LINE-1 was evaluated by bisulfite-PCR and pyrosequencing [28] in all tumors and in twenty-five samples of normal colonic mucosa. Thirteen of these samples were derived from normal tissue at the resection margins of 13 patients with sporadic CRCs. The remaining normal specimens were obtained from 12 individuals who had undergone surgery for ischemic colorectal disease or for diverticulitis without a personal history of colorectal cancer. Bisulfite treatment of genomic DNA converts all unmethylated cytosines into thymine while methylated cytosines remain unchanged. All the cytosine residues unconverted in the sequence represent methylated cytosines in the genome. In this method, 1.5 mg of DNA was denatured in 50 mL of 0.2 M NaOH for 10 min at 37°C. Then, 30 mL of freshly prepared 10 mM hydroquinone and 520 mL of 3 M sodium bisulfite at pH 5.0 were added and mixed. The samples were incubated at 50°C for 16 h. The bisulfite-treated DNA was purified using Wizard DNA Clean-Up System (Promega).

LINE-1 assay was designed toward a consensus LINE-1 sequence (GenBank accession number X58075) and allowed to quantify the percentage of 5-methylated cytosines (%5mC) in five consecutive CpG sites. PCR was performed in a 50 *μ*L reaction volume that included 2 pmol of forward primer 5′-GAGTTAGGTGTGGGATATAGT-3′, 2 pmol of reverse biotinylated primer 5′-CAAAAAATCAAAAAATTCCCTTCCC-3′, 5 *μ*L of bisulfite-treated genomic DNA 1.25 units of GoTaq DNA polymerase, 1X GoTaq Flexi Buffer (Promega, Madison, WI, USA), and 200 *μ*M dNTPs. Thermal cycling conditions were 3 min at 95°C, 45 cycles at 95°C/25 s, 50°C/25 s, and 72°C/25 s, followed by a final extension at 72°C for 5 min. Pyrosequencing was performed on PCR product with bound LINE-1 sequencing primer 5′-GGTGTGGGATATAGTT-3′, according to the protocol reported above. Fully methylated DNA (CpGenome Universal Methylated DNA, Millipore, Billerica, MA, USA) and unmethylated DNA (CpGenome Universal Unmethylated DNA, Millipore, Billerica, MA, USA) were used as positive and negative controls for optimizing the assay.

### 2.9. Statistical Analysis

Univariate comparisons of continuous data were carried out using Student's *t*-test and discrete variables were compared with *χ* ^2^ test or Fisher's exact test. The association between discrete outcome and continuous predictor was evaluated with a logistic regression model. All comparisons were two-sided and a *p* value <0.05 was considered to be significant.

## 3. Results

### 3.1. Patients Features

We recruited a total of 38 patients with adenocarcinoma in the colon-rectum diagnosed before the age of 40 years, but the biological material was available for 33 patients. Clinicopathological features of the 33 colorectal adenocarcinomas and patients are shown in Table 1. The mean age of disease onset was 35 years. We observed that the male gender was approximately 3 times more frequent than female (76% versus 24%), tumor location was preferentially the left colon (52%), and C and D stages of Dukes (37% and 27%, resp.) were more frequently represented. Fifty-eight percent of early onset tumors were well or moderately differentiated. Family history of colorectal cancer was present in 8 (24%) patients: 2 with Bethesda criteria (patients with a first-degree relative affected by colorectal cancer), 5 who fulfilled the Amsterdam II criteria, and 1 with Familial Adenomatous Polyposis. Clinical features of Cowden/Bannayan [29] and Peutz-Jeghers syndromes [30] were not identified in any patients.

### 3.2. MMR Deficiency and Somatic Mutations of* KRAS* and* BRAF* Genes

MMR deficiency was evaluated by both immunohistochemistry and MSI analysis in all 74 colorectal cancers. MMR deficiency was defined as loss of protein expression in any of the MMR proteins and/or having a MSI tumor. Seven out of 33 (21%) early onset tumors showed MSI and loss of expression of MMR proteins (4 for MLH1/PMS2 and 3 for MSH2/MSH6). By family history, 5 patients (≤40 years) with MMR deficient tumors fulfilled clinical features of Lynch syndrome and 2 the Bethesda Criteria. Four out of 41 (10%) tumors in the control group (4 for MLH1/PMS2) were MMR deficient. These 4 tumors diagnosed over the age of 60 years showed somatic* MLH1* hypermethylation and V600E mutation in* BRAF* gene. No V600E mutations were detected in MSS tumors with clinical onset at advanced age or in early onset colorectal cancers. We found somatic* MSH2* methylation in only one case (patient with germinal* EPCAM* deletion) among 7 young patients showing MSI and loss of MSH2/MSH6 protein expression.* KRAS* mutations were found in 10 early onset tumors and in 4 cases of the control group. Thus, tumors of juvenile cases showed more often somatic* KRAS* mutations (30% versus 10%). Molecular results in the investigated patients are summarized in Table 2.

### 3.3. Germline MMR,* APC*, and* MUTYH* Mutations

We identified 6 cases with sequence variants in either* MLH1* (3 cases),* MSH2* (1 case),* EPCAM* (1 case), or* APC* (1 case) (Table 3). For* MLH1*, we detected one insertion mutation (p.Arg497ProfsX6) and two missense mutations (p.Leu749Pro and p.Glu663Asp). These two missense variants were classified as Class 5 mutations (>99% likelihood of pathogenicity) by Plon et al. [31]. For* MSH2*, we found one nonsense mutation (p.Phe294X). Moreover, we identified a large deletion of exons 8 and 9 in* EPCAM* gene, without involvement of* MSH2* promoter, using the P003-B2 MLPA kit. This finding was confirmed by the P072-B1 MLPA kit (MRC-Holland). Only one patient was affected by Familial Adenomatous Polyposis and showed an insertion mutation in* APC* gene at exon 15 (p.Arg924SerfsX16). Germline analysis of* MUTYH* gene was carried out in the whole group of young patients, but no monoallelic or biallelic alteration was detected.

### 3.4. Gene-Specific DNA Methylation

**Figure 1:**
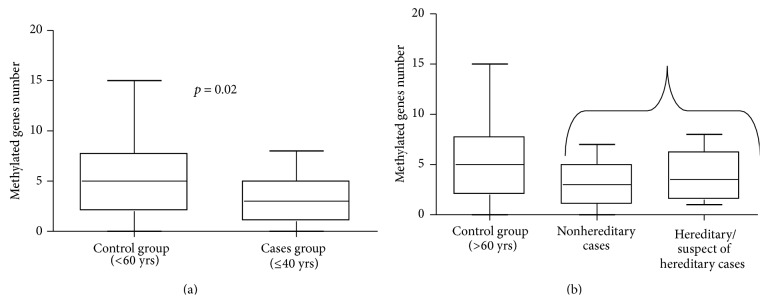
Results of methylation status in young group and in elderly group. In (a), we observed a significant difference in methylation pattern between patients under 40 yrs and patients over 60 yrs. The mean number of methylated genes in the control group is higher than average number in the cases group. In (b), we observe no statistical difference between control, young, and hereditary cases, but the difference remained significant between patients under 40 yrs and patients over 60 yrs.

MS-MLPA assay was employed to evaluate the hypermethylation profiles relative to tumors from early onset patients (*n* = 33) and control group (*n* = 41). The average number of methylated genes was significantly higher in the control group compared with the young group (5.025* versus* 3.3, resp.) (*p* = 0.02, Figure 1(a)). By contrast, no significant differences were observed between young and hereditary/suspect of hereditary cases (Figure 1(b)).

**Figure 2:**
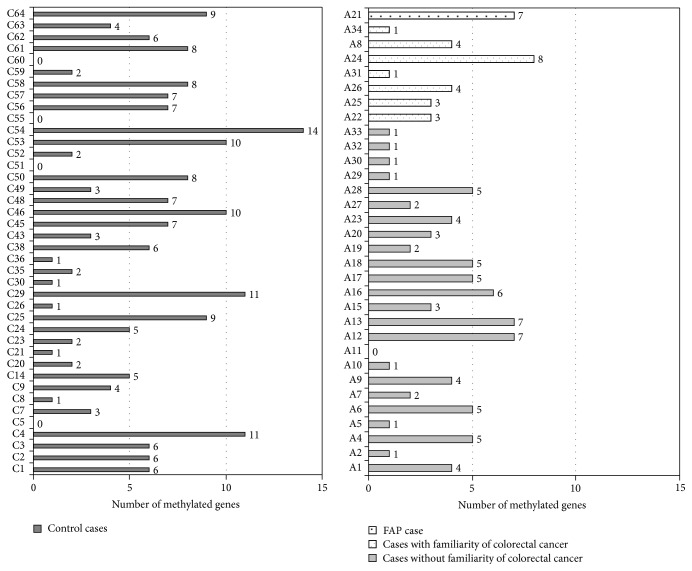
Number of methylated genes in control cases and in early onset colorectal cancer.

In the control group, we observed two main tumor sets on the basis of the degree of methylation. The first group consisted of 7 CRCs (17% of cases) showing high levels of gene methylation, involving a mean percentage of 28% of the promoters examined (ranging from 24% to 37%). The second group included the remaining 34 CRCs showing absent or low level methylation involving a mean percentage of 9% of the genes analysed (ranging from 0% to 20%) (Figure 2).

The seven CRCs exhibiting extensive gene methylation included all the cases showing MSI and* BRAF *mutation and* MLH1* methylation.

**Figure 3:**
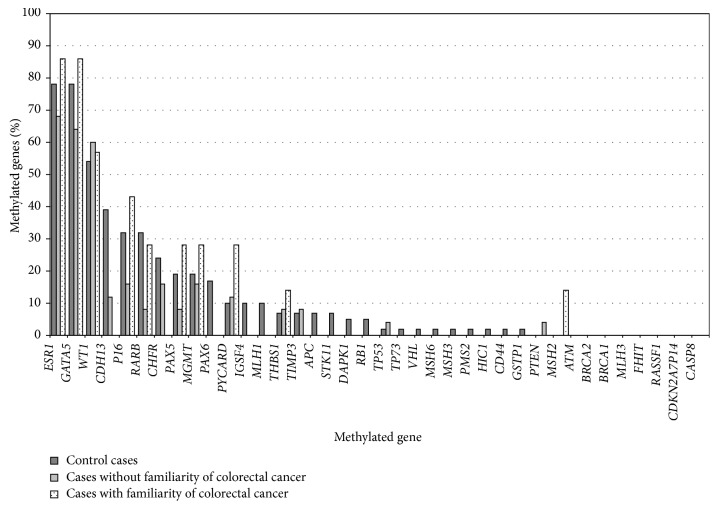
Hypermethylation frequency in single genes.

On the other hand, in the group of early onset tumors, we did not detect any case with extensive gene methylation. No methylation for the* MMR* genes was found in these tumors, with the exception of the case with* EPCAM* gene mutation (A34), which was methylated in the* MSH2* gene. Evaluating the hypermethylation frequency in single genes, no significant differences were observed comparing early onset patients and control group. By contrast, a very high frequency of methylation was detected in* ESR1*,* GATA5*, and* WT1* genes in both groups of the familiarity (Figure 3).

### 3.5. LINE-1 Hypomethylation

We used the quantitative bisulfite pyrosequencing to determine the methylation status of LINE-1 repetitive sequences in all CRCs compared to twenty-five samples of normal colonic mucosa. In normal samples, LINE-1 methylation levels were high (average 60.78% ± 0.4%) and very similar to those commonly observed in peripheral blood cells from normal individuals [32]. By contrast, LINE-1 methylation levels in CRCs were significantly lower than in normal samples (mean 55.4% ± 0.86; *p* < 0.024).

**Figure 4:**
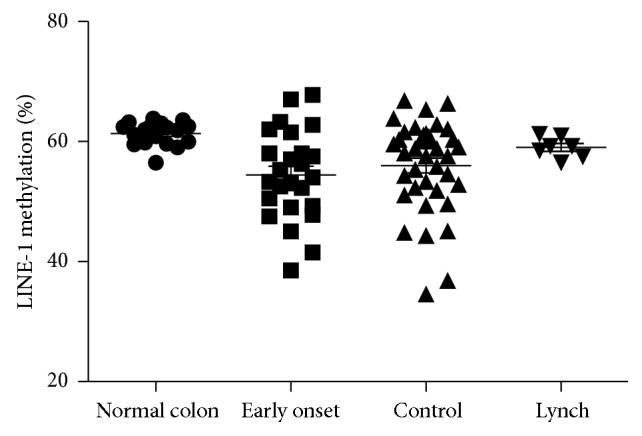
LINE-1 hypomethylation in early and late onset patients.

Mean LINE-1 methylation levels in the three study groups of colorectal cancers were early onset, 54.3%; late onset, 55.9%; Lynch syndrome, 59.0% (Figure 4). The difference of LINE-1 hypomethylation in early onset colorectal cancer was not significant when compared to late onset ones. Interestingly, in the Lynch syndrome tumors LINE-1 methylation levels were higher than early onset and late onset groups and similar in the mean percentage of normal mucosa. However, this difference did not reach statistical significance because of the small number of Lynch tumors.

## 4. Discussion

In this study we have assessed the clinicopathological, molecular, and familial features of 33 early onset colorectal cancers (≤40 years). We showed that the frequency of known hereditary colorectal cancer syndromes in this population was 18%. This cohort disclosed a molecular profile of* MMR* deficiency characterised by germline mutations in* MLH1*,* MSH2*, and* EPCAM* genes, thus confirming that Lynch syndrome is the most frequent cause of hereditary colorectal cancer in young patients [12, 19].

Previous studies have revealed that colorectal cancer diagnosed at early ages had a high probability of showing* MMR* deficiency, ranging from 26% to 73% [11]. The reason for this wide range could be explained by the different age thresholds (from 24 to 50 years) and by diverse panels of proteins analysed by IHC (usually restricted to MLH1 and MSH2). Moreover, the majority of studies have analysed either Lynch syndrome or sporadic colorectal cancer and it is known that the rate of MSI is much lower in the latter. Our study, based on a population based approach of colorectal cancer developed ≤40 years recruited from the specialised Colorectal Cancer Registry of Modena, showed that the frequency of MMR deficiency was 21%. Although we identified 7 tumors with MMR deficiency, we detected only five patients with pathogenetic germline mutations in* MMR* genes. Possible causes for the lack of identified constitutional mutations could be low sensitivity of analytical methods or genetic events [33] that affect both alleles of a* MMR* gene. We did not identify any carrier of* MSH6* germline mutations. Recent studies have shown that the average age of colorectal cancer onset in* MSH6* mutation carriers has been estimated to be around 50 years, while* MLH1* and* MSH2* carriers are diagnosed on average 10 years earlier [34]. Moreover, mutations in the* MSH6* gene have also been linked to a lower risk of colorectal cancer and a higher risk of endometrial carcinoma [34]. Although germline mutations of* MSH6* gene in early onset colorectal cancer have been reported [35], this difference in age of onset and associated risk may explain why* MSH6* mutations constitute a minor fraction of cases.

In summary, as suggested by Jasperson et al. [11], the study of family cancer history, MSI, and IHC analyses followed by germline genetic testing represent an effective procedure for the diagnosis of Lynch syndrome in early onset cases.

Some population based studies showed that ~30% of biallelic* MUTYH* mutation carriers develop a colorectal cancer in the absence of a polyposis phenotype [13, 16, 35]. Accordingly, it has been suggested that* MUTYH* testing should be considered in early onset colorectal cancer patients with intact DNA MMR, regardless of family history or number of colonic polyps [13]. Giráldez et al. detected 2.8% of biallelic* MUTYH* mutations in a cohort of 140 patients with colorectal cancer diagnosed before the age of 50 [36]. In our study we performed systematic whole-gene sequencing and did not find biallelic or monoallelic* MUTYH* mutations. These negative results could be explained by the limited number of investigated cases.

We identified only one pathogenetic mutation of* APC* in a 34-year-old patient with colorectal cancer and polyposis. The literature describes that Familial Adenomatous Polyposis is responsible for less than 1% of all colorectal cancers [37] and that the mean age for colorectal cancer development in this group of individuals is approximately 39 years [38], suggesting that also* APC* gene is implicated in cancer occurring at an early age.

In agreement with that reported in literature, we observed that 82% of early onset CRCs were not associated with known hereditary CRC syndromes [36]. This subset of tumors was mainly characterized by distal location, advanced stage, and predominance of the male gender as well as other investigators supported [39]. A previous study according to Ahnen et al. reported that cancer-specific survival in patients with young onset CRC is comparable to that of patients with late onset cancer [5, 38]. Moreover, we observed a similar frequency of somatic mutations for* KRAS *oncogene to overall sporadic colorectal cancers that correspond to 40% [40]. Regarding* BRAF* gene, a recent study suggests that* BRAF* mutations occur in 10–20% of sporadic colorectal cancer and are closely associated with the* MLH1* methylation. In our young patients, no* BRAF* mutations or* MLH1* promoter methylation was detected.

A second important aspect of our work concerns the analysis of aberrant hypo- and hyper-DNA methylation profiles of early onset CRCs compared with late onset CRCs. Both of these alterations are widely accepted as potential source of early biomarkers for diagnosis and prognosis in CRCs. CIMP phenotype, which accounts for almost 30–40% [41], has rarely been evaluated in early onset CRCs. In our work, no case with extensive gene methylation was observed among early CRCs and the average number of methylated genes was significantly lower in these tumors (both hereditary and nonhereditary CRCs) compared with the control group.

These results are in agreement with previous works that reported low levels of gene hypermethylation in Lynch syndrome cases [42, 43] as well as in nonhereditary early onset CRCs [19].

In our study, extensive gene hypermethylation was observed only in the late onset CRCs, accounting for 17% of these cases. In agreement with literature, this subset of CRCs frequently showed MSI and* BRAF* mutation and* MLH1* methylation [41, 43].

For the first time, we demonstrated that hypermethylation of three genes, namely,* ESR1*,* GATA5*, and* WT1*, was very common both in early onset (hereditary and nonhereditary tumors) and in late onset CRCs. Although these results need to be validated with further studies, our data have important clinical implications suggesting the usefulness of aberrant gene methylation analysis for the early detection and risk assessment of CRC, without using age at onset as a differential criterion. Promoter methylation analysis of serum and stool DNA has the potential to be used as a noninvasive test for the early diagnosis of CRC [44]. However, accurate selection of methylation markers is crucial for sensitive and specific detection of CRC as* de novo* methylation is also associated with aging [45].* ESR1* is a well-known “type A” (age related) gene because its hypermethylation is demonstrated in both normal colorectal mucosa and CRCs, proportional to tissue age. By contrast,* GATA5* methylation has been reported as a suitable marker for early diagnosis of CRC [46] and its methylation is observed in colorectal adenomas but not in inflammatory colorectal tissues.

Moreover, our analysis has highlighted the potential utility of the* WT1* gene as an early diagnostic marker of CRC. To date, only a few studies have investigated* WT1* methylation, confirming our data of widespread methylation of this gene in CRCs [47, 48]. In our opinion, this finding deserves to be explored further, especially with respect to the aberrant mechanisms of loss of imprinting of 11p15 described in CRCs and the possible link between* WT1* methylation and the upregulation of* IGF2* transcription [49].

Genome-wide hypomethylation is also reported as an early event in CRC and it has been associated with the activation of protooncogenes (i.e.,* MET*) [50] and the presence of chromosomal instability [51]. Recently, Antelo et al. [39] found significantly lower levels of LINE-1 methylation in early onset CRCs compared to late onset CRCs, suggesting that a high degree of LINE-1 hypomethylation is a unique feature of CRCs in young patients. At variance, our study does not confirm these previous findings, demonstrating very similar LINE-1 methylation levels in early onset and in late onset CRCs, with a normal distribution of LINE-1 values in both subsets of tumors. In our opinion, several factors may explain this discordance. Firstly, since the degree of LINE-1 demethylation prognosis is linear in relation to TNM-stage progression and this marker is a strong independent factor for poor prognosis [52], the evaluation of tumor stage is crucial when comparing different subsets of CRCs. The second important point regards the tumor location because lower levels of LINE-1 methylation have been reported in distal compared with proximal CRCs [39]. Antelo et al. examined two independent cohorts of CRCs developed ≤50 years including mainly advanced and distal CRCs without a matched selection of the late onset CRCs. For these reasons we designed a case-control study in which every early onset CRC was matched with a late onset CRC for sex, location, and stage. Finally, technical reasons may not be excluded, although the same methodological approach has been used in the two studies and very similar LINE-1 methylation levels were observed considering normal colorectal mucosa and MSI CRCs (both hereditary and sporadic tumors).

Moreover, Antelo et al. reported a similar result about higher levels of LINE-1 methylation in Lynch syndrome tumors than in group of early onset CRC. For this reason, it is maybe not optimal to use this testing for detection of early onset CRC, whereas Lynch syndrome is the most common hereditary CRC in young patients. Although our series of early onset CRCs is small and the present results are not conclusive about this issue, we believe that the strong positive association between LINE-1 hypomethylation and early onset CRC previously reported needs to be reconsidered through future larger case-control studies.

## 5. Conclusion

The results of this study can be summarised as follows.

First, Lynch syndrome is the most frequent cause of hereditary colorectal cancer in young patients; family cancer history, MSI, and IHC analyses followed by germline genetic testing represent the most appropriate procedure for Lynch syndrome diagnosis in early onset colorectal cancer. Second, early onset colorectal cancers with MMR deficiency were clinically and pathologically indistinguishable from colorectal MSS carcinomas. Third, epigenetic events (hyper- and hypomethylation) are not closely associated with early onset colorectal cancer. Finally, our study emphasises that the genetic basis in the majority of early onset colorectal carcinomas remains unknown. Further studies of the whole exome of this genetically undefined group of early onset colorectal tumors will need to elucidate possible pathogenetic mechanisms.

## Figures and Tables

**Table 1:** Clinicopathological features of 33 colorectal adenocarcinomas (and patients) developed ≤40 years (1984–2008) compared to patients with cancer onset >60 years.

	Technical details	Patients with onset ≤40 years, *N* = 33 (%)	Patients with onset >60 years, *N* = 41 (%)
Age of onset of disease	Min Max Mean	11 40 35	61 85 73

Sex	Female Male	8 (24) 25 (76)	11 (27) 30 (73)

Tumor location in the large bowel	Right colon Left colon Rectum	7 (21) 17 (52) 9 (27)	12 (29) 19 (47) 10 (24)

Stage (Dukes)	A B C D	5 (15) 7 (21) 12 (37) 9 (27)	7 (17) 9 (22) 14 (34) 11 (27)

Tumor differentiation	Low-grade High-grade Mucinous	19 (58) 8 (24) 6 (18)	31 (76) 8 (19) 2 (5)

**Table 2:** Molecular features of colorectal carcinomas in cases and in the control group.

Technical detail	Patients with onset ≤40 years		Control group >60 years, *N* = 41 (%)
	*N* = 33		
	Nonhereditary cases, *N* = 25 (%)	Hereditary/suspect of hereditary cases, *N* = 8 (%)	
MMR alterations			
MSI and no expression MMR proteins	0	7 (88)	4 (10)
Methylation			
*MLH1 *or *MSH2 *promoter hypermethylation	0	1 (13)	4 (10)
Somatic mutations			
*KRAS* mutation	9 (36)	1 (13)	4 (10)
*BRAF* mutation	0	0	4 (10)
Germline mutations			
*MMR *	0	5 (63)	0
*APC*	0	1 (13)	0
*MUTYH *	0	0	0

**Table 3:** Constitutional mutations in early onset colorectal cancer.

	Cases	Gene	Mutation
Lynch syndrome	5	*MLH1 *(3)	c.2246T>C; p.Leu749Pro
			c.1489dupC; p.Arg497ProfsX6
			c.1989G>T; p.Glu663Asp
		*MSH2 *(1)	c.881_882delTT; p.Phe294X
		*EPCAM* (1)	Del ex 8-9 and seq in 3′ to +3 kb

Familial Adenomatous Polyposis	1	*APC *	c.2771_2772insT; p. Arg924SerfsX16
